# 500 mg as bolus followed by an extended infusion of 1500 mg of meropenem every 8 h failed to achieve in one-third of the patients an optimal PK/PD against non-resistant strains of these organisms: is CRRT responsible for this situation?

**DOI:** 10.1186/s13613-020-00777-2

**Published:** 2020-12-03

**Authors:** Patrick M. Honore, Leonel Barret Gutierrez, Luc Kugener, Sebastien Redant, Rachid Attou, Andrea Gallerani, David De Bels

**Affiliations:** ICU Dept, Centre Hospitalier Universitaire Brugmann-Brugmann University Hospital, Place Van Gehuchtenplein, 4, 1020 Brussels, Belgium

We read with great interest the recent paper by Kothekar et al. who conclude that in patients with severe sepsis or septic shock, extended infusions (EI) of 1000 mg of meropenem over 3 h, administered every 8 h on the first and third days, provided adequate coverage against sensitive strains of Enterobacteriaceae, *Pseudomonas aeruginosa* and *Acinetobacter baumannii* [1]. However, this dosing regimen failed to achieve a fraction of time (fT) > 4 μg/mL > 40 for activity against non-resistant strains of these organisms in more than one-third of patients [1]. A bolus of 500 mg followed by EI of 1500 mg every 8 h was predicted to achieve this target in all patients [1]. The question is why was this the case in this study. We would like to comment. Though the study excluded at baseline patients with calculated creatinine clearance < 50 mL/min and those not expected to survive for 72 h, the cohort of patients included in the study had severe sepsis, with a mean SOFA score at day 1 of 7.35 ± 3.62 and 60% required inotropes. As such, we would expect a higher likelihood of acute kidney injury (AKI) and the need for renal replacement therapy (RRT) in this cohort. Nearly half of critically ill patients, especially those with septic shock, have or develop AKI and 20–25% need RRT within the first week of admission to intensive care [2]. Losses of meropenem are significant by convection and dose adaptations are necessary [3]. According to a population PK/PD model of meropenem developed in critically patients undergoing continuous RRT (CRRT), Isla et al. [4] recommended continuous infusion (CI) for treatment of pathogens with a MIC ≥ 4. In that study, meropenem was significantly eliminated by CRRT, necessitating steady-state doses of 1 g every 8 h to maintain concentrations active against more resistant organisms [4]. Because the stability of meropenem reconstituted in solution is influenced by storage temperature [5], it is advised to infuse 2 g meropenem for 8 h, 3 times daily to cover a 24 h period [3]. It stands to reason as in the Kothekar et al. paper that if drug dose adaptation was not done in CRRT patients and CI was not used in cases of pathogens with a MIC ≥ 4, some of the patients may have been underdosed, even with 1 g every 8 h. Again, in the Kothekar et al. paper it would be interesting to know the proportion of patients in the study who received CRRT, especially amongst the patients who failed to achieve adequate PK/PD.

## Data Availability

Not applicable.

## Abbreviations

EI: Extended infusions
fT: Fraction of time
SOFA: Sequential Organ Failure Assessment
RRT: Renal replacement therapy
AKI: Acute kidney injury
PK/PD: Pharmacokinetics/pharmacodynamics
CRRT: Continuous renal replacement therapy
CI: Continuous infusion
MIC: Minimum inhibitory concentration
